# The new progress in cancer immunotherapy

**DOI:** 10.1007/s10238-022-00887-0

**Published:** 2022-09-15

**Authors:** Ajmeri Sultana Shimu, Hua-xing Wei, Qiangsheng Li, Xucai Zheng, Bofeng Li

**Affiliations:** 1 Department of Medical Oncology, Division of Life Sciences and Medicine, The First Affiliated Hospital of USTC, University of Science and Technology of China, Hefei, 230001 China; 2 Department of Laboratory Medicine, Division of Life Sciences and Medicine, The First Affiliated Hospital of USTC, University of Science and Technology of China, Hefei, 230001 China

**Keywords:** Cancer, Immunotherapy, Immune checkpoint, Siglec, SIRP-α, NK cells

## Abstract

The cross talk between immune and non-immune cells in the tumor microenvironment leads to immunosuppression, which promotes tumor growth and survival. Immunotherapy is an advanced treatment that boosts humoral and cellular immunity rather than using chemotherapy or radiation-based strategy associated with non-specific targets and toxic effects on normal cells. Immune checkpoint inhibitors and T cell-based immunotherapy have already exhibited significant effects against solid tumors and leukemia. Tumor cells that escape immune surveillance create a major obstacle to acquiring an effective immune response in cancer patients. Tremendous progress had been made in recent years on a wide range of innate and adaptive immune checkpoints which play a significant role to prevent tumorigenesis, and might therefore be potential targets to suppress tumor cells growth. This review aimed to summarize the underlying molecular mechanisms of existing immunotherapy approaches including T cell and NK-derived immune checkpoint therapy, as well as other intrinsic and phagocytosis checkpoints. Together, these insights will pave the way for new innate and adaptive immunomodulatory targets for the development of highly effective new therapy in the future.

## Introduction

The immune system is made up of a complex network of interconnected mechanisms that work together to provide an adaptive immune reaction against carcinogenesis and infection while maintaining immune tolerance to self-antigens [1]. On a cellular level, cancer is caused due to the accumulation of numerous genetic alterations that result in the breakdown of normal regulatory systems. Nowadays, immunotherapy is a well-known treatment strategy for cancer that can enhance the existing anti-tumor response so that immune cells can restrict the growth of malignant cells. Again, immunotherapy can effectively target the cancer cells while causing minimal adverse effects [2, 3]. Moreover, to provide protective immunity against the tumor cells, a range of successive events take place in order. For example, the discharge of neo-antigens from malignant tumors in the first step is recorded by the dendritic cells [4]. The second step is known as the processing step where the release of pro-inflammatory cytokines and factors triggers the activation of effector T cells by presenting the dendritic cell-mediated captured antigens to T cells [5]. Then, the recognition and killing of cancer cells occur through the infiltration of activated T cells such as CD8^+^ effector T cells after trafficking into the tumor mass. After the death of cancer cells, more tumor-related proteins are secreted to increase the efficiency of successive anti-tumor immunological cycles [6]. Also, immunogenic cancer cells are killed by effector CD8^+^ T cells due to the expression of MHC-1 molecules on their cell surface, while NK cells can kill non-immunogenic cancer cells that do not contain MHC-1 molecules. Based on this understanding, cancer vaccines and immune checkpoint therapies are being created to attack immunogenic tumors that produce antigenic peptides to naive CD8^+^ T cells, whereas transfection of genetically modified T cell treatment can identify tumor cell-derived surface proteins that directly have evolved to target non-immunogenic tumors [7]. Therefore, it is critical to select an appropriate therapeutic technique depending on the tumor’s features to improve outcomes.

However, tumor progression occurs through the continuous development of an immune-suppressive channel by cancer cells that are mainly responsible for activating a variety of regulatory processes that allow immune evasion [8, 9]. Indeed, cancer cells can evade the immune-surveillance pathway by displaying a wide range of co-inhibitory components such as programmed cell death ligand-1/2 (PD-L1/2), cytotoxic T lymphocyte antigen-4 (CTLA4), T cell immune receptor with Ig and ITIM domains (TIGIT), T cell immunoglobulin and mucin-domain containing-3 (TIM-3), and lymphocyte activating antigen-3 (LAG-3) [10]. However, the proliferation of diverse cell types that express inhibitory chemicals, such as TAMs (tumor-associated macrophages), Tregs (regulatory T cells), and MDSCs (myeloid-derived suppressor cells) also promotes the escape of effective immune response. On the other hand, the response of effector CD4^+^ and CD8^+^ T cells can be reduced by the interactions with these co-inhibitory proteins [11, 12]. Furthermore, cancer cells can potentially avoid immune response by down-regulating MHC class I expression, resulting in a poor response of effector CD8^+^ cytotoxic T cells against tumors [13]. To suppress protective immunity, cancer cells also produce IL-10 (interleukin-10) and different growth factors such as VEGF (vascular endothelial growth factor) and TGF (transforming growth factor) [14–16].

Recently, numerous immune treatment methods, such as immune checkpoint blockade of co-inhibitory receptors using anti-PD1/PDL1 and anti-CTLA4, dendritic cell vaccines, and cytokine therapy, have been designed and therapeutically used to address tumor cell immune evasion [17]. Among these methods, ICB has made a breakthrough in developing long-lasting and effective anti-tumor immunity against melanoma, non-squamous non-small cell lung cancer, and metastatic bladder cancer [18, 19]. Another type of cancer immunotherapy technique is known as ACT (adoptive cell transfer) which employs T cell receptor (TCR)-modified T cells or tumor-infiltrating T cells (TILs) or genetically engineered chimeric antigen receptor-specific T cells (CAR-T) to treat the cancerous cell more precisely and effectively [20]. Furthermore, combining effective treatments can improve the performance of immunotherapy, resulting in long-lasting anti-tumor immunomodulatory responses. In cancer immunotherapy, it is critical to understand the potential features of tumor immune-regulatory pathways so that they can provide an efficient immune system response [21, 22]. To enhance anti-tumor immune response, the immunotherapy technique exerts the identification and programmed cell death mechanisms of malignant cells. Although immunotherapy is regarded as a promising therapeutic approach to treat cancer patients, there is still a need for successful new treatments because immunotherapy approaches are limited to a subset of cancer types and individuals with minor or no therapeutic benefits. Therefore, this review aimed to briefly elucidate the existing molecular and cellular mechanisms by focusing on how tumor cells escape the immune surveillance on T cells, macrophages, and NK cells, which will then lead to the development of a novel therapeutic approach to promote the anti-tumor immunity within close future.

## T cell immune checkpoints-based immunotherapy

Immune checkpoints are important regulatory pathways in the immune system that are triggered by ligand-receptor interactions. Besides, they have a crucial role in the development of an effective immune response to eliminate infectious particles and tumor cells but no response against the self-antigens [5]. Generally, they can exhibit both co-stimulatory substances such as CD27, CD28, CD137, ICOS, 4-1BB, and OX-40 enact T cells to activate T cells and co-inhibitory molecules like PD-1, CTLA-4 to suppress the activity of T cells. Some of the inhibitory checkpoints are overexpressed within the tumor microenvironment to promote tumor cells-mediated immunosuppression.

To trigger therapeutic anti-tumor immunity, co-inhibitory immune checkpoint molecules can be blocked by antibodies or altered by recombinant forms of ligands or receptors [22]. The first immune therapeutics approved by the US Food and Drug Administration were antibodies against CTLA-4. Recently, more attention among the many other promising approaches due to its remarkable outcomes has been received [23]. Besides CTLA-4, now several immune checkpoint receptors (ICRs), such as PD‐1, LAG3, TIM-3, B7‐H3, and diacylglycerol kinase α, have been also identified to treat cancer patients effectively [24, 25]. Additionally, the expression of these co-inhibitory receptors has been detected in tumor-infiltrating CD4/CD8 T_eff_ cells and Tregs which are also involved to create tumor evasion [26]. Therefore, the targeting of CTLA-4 and PD-1 negative immune checkpoints-based immune suppression pathway along with some other important regulatory checkpoints such as Siglec factors and CD47-SIRPα may contribute to the upgrade of immune responses against cancer cells.

### CTLA-4

CTLA-4, also known as CD152, is the first detected co-inhibitory checkpoint receptor (ICR) that normally expressed on T cells and is linked to the suppression of endogenous anti-tumor immunity mediated by T cells [27, 28]. Foxp3 and NFAT (nuclear factor of activated T cells) are the two regulatory factors that play an important role in the transcription of CTLA-4, which controls immunologic tolerance [29]. CTLA-4 deficiency in FoxP3^+^ cells impairs the inhibitory functions of Treg cells which are considered the biggest obstacle in immunotherapy targets due to their anti-tumor suppressing nature [30]. According to Buchbinder & Desai’s findings, CTLA-4 and CD28 have two similar ligands to outcompete each other including B7.1 (also known as CD80) and B7.2 (also known as CD86), whereas CTLA-4 has a more affinity to bind with those ligands than CD28 [31]. On the other hand, the interaction of CTLA-4 with its ligands not only suppresses T cells but also reduces the expression of dendritic cells-mediated immunologic signals [32]. However, the survival and activation of T cells occur when a high proportion of CD28 molecules are present in the tumor microenvironment to trigger the secretion of IL-2 cytokine which is responsible for the rise in metabolic rate [33]. Furthermore, various investigations on T cell signaling and activation have demonstrated that active SHP2 and PP2A proteins have the potential to neutralize the signaling kinases by stimulating CD28 and TCR [34]. When CTLA-4-derived inhibitory signals are significantly transferred into T cells, both CD80 and CD86 ligands are removed from the surface of APCs by disrupting their interaction with CD28 and TCR [35]. However, Eggermont et al. found that lowering the Tregs effective performance can increase the susceptibility to autoimmune diseases in patients with multiple sclerosis, type II polyglandular syndrome, type 1 diabetes, rheumatoid arthritis, psoriasis, and myasthenia gravis in comparison with healthy people [36]. In case of cancer immunotherapy, the use of monoclonal antibodies of CTLA-4 could increase T cell proliferation and their cytokine production. For example, the monoclonal antibodies ipilimumab and tremelimumab have been recently applied for the CTLA-4 blocking strategy which results in the growth of blood cells in the immune system causing cancer cells death [23]. Again, combining CTLA-4 therapeutic inhibition with cancer vaccines may provide effective anti-tumor immunity through the efficient induction of immune response against low immunogenic tumor cells [37].

### PD-1

PD-1 is another negative immune checkpoint receptor that is expressed on a variety of immune cells such as activated T cells and B cells, myeloid cells, NK cells, dendritic cells, and monocytes [38]. In addition, it belongs to the immunoglobulin superfamily as a monomer and has a vital suppressive role against T cell activation, similar to CTLA-4 [39]. After interacting with B7 family ligands, including PD‐L1 (B1‐H1) and PD‐L2 (B7‐DC), PD-1 inhibits the activation of T lymphocytes by limiting ZAP70/PI3K enzyme kinase activity via SHP2 phosphatase [40]. Active CD8^+^ T lymphocytes express PD-L1, while the expression of PD-L2 is primarily restricted to immune system cells. Like CTLA-4, it also stimulates Treg cell signaling, which is important for triggering immune suppression of effector T cells that control immune response homeostasis [41]. PD-1 ligands are more interesting than PD-L2 ligand because tumor cells can express PD-L1 as a result of inflammatory cytokines and oncogenic signaling pathways [32]. Furthermore, PD-1 interacts with its ligands immediately after TCR stimulation, resulting in phosphorylation of tyrosine receptors based on the inhibition of immune receptor tyrosine‐based inhibition motif [ITIM] and the switching of immune receptor tyrosine‐based switch motif [ITSM] [42].

To boost the activity of NK cells and PD-1^+^ B cells-mediated antibody production within the tumor microenvironment, blocking of the PD-1 pathway is used as a first method to improve the activity of effector T cells against tumor cells [43–45]. It is found that Treg cells are the primary target of PD-1 blockade method which can be detected in the peripheral blood in a study of stage IV melanoma patients [45]. Two studies have recently reported that combining PD-1/PD-L1 therapies with the cytokine TGF-β can enhance the efficacy of anti-tumor response through CTL infiltration to prevent the growth and metastatic spread of both murine EMT6 breast mammary carcinoma and orthotopic colorectal cancer vaccination models [22, 46]. Taken together, these findings might provide the foundation for combining the PD-1 blocking pathway with other inhibitors to improve anti-tumor effector actions [47]. Several monoclonal antibody factors are recently used to block the PD-1 pathway, including FDA-approved nivolumab (a fully humanized IgG4) for treating melanoma and non-small cell lung cancer, another IgG4-based antibody, pembrolizumab, for treating skin cancer, and durvalumab (MEDI4736), which blocks PD-L1 and binds to PD-1 as well as CD80 [23].

## Siglec-based immunotherapy

The development of acquired resistance in FDA-approved CTLA-4 and PD-1 immunosuppressive antibodies triggers to search for additional more effective therapeutic strategies so that the efficacy of PD-1 or PD-L1 blockade pathway can enhance T cell immune response in contrast to tumor cells [48]. To achieve this demand, another type of immune checkpoint receptors like Siglecs is needed to be targeted to promote innate and adaptive immune cells-mediated anti-tumor immunity. The Siglecs (sialic acid-binding immunoglobulin-like lectins) are type-1 immunoglobulin-like transmembrane immune cell receptors that bind a wide range of sialic acids ligands by using an amino-terminal V-set immunoglobulin domain and also display variable numbers (16 in the case of sialoadhesin) of C2-set immunoglobulin domains [49, 50]. They can be divided into two groups such as CD33 related Siglecs (Siglec-3 (CD33), Siglec-5, Siglec-6, Siglec-7, Siglec-8, Siglec-9, Siglec-10, Siglec-11, Siglec-14, and Siglec-16) and conserved Siglecs (Siglec-1, Siglec-2, Siglec-4, and Siglec-15) based on sequence similarity and evolutionary conservation. By contrast to conserved Siglecs, CD33-related Siglecs have highly similar (~ 50–99%) sequences in their extracellular domains beyond their different composition property and also have one or more intracellular ITIMs which have the potential to suppress activation signals of immune cells through the recruitment of tyrosine and inositol phosphatases(Crocker et al., 2007) [51]. There are nine CD33-related Siglecs in humans, and they are mostly expressed by mature innate immune cells such as neutrophils, eosinophils, monocytes, macrophages, NK cells, DCs, and mast cells. Self-associated molecular patterns (SAMPs) are developed when Siglecs bind to diverse sialoglycan ligands, and individual Siglecs have variable binding preferences for sialoglycan ligands [52, 53]. Current studies revealed that the inhibitory receptors of CD33r Siglecs can form a bridge between immune cells and tumor cells via a sialic acid-dependent mechanism [54]. Therefore, targeting the sialoglycan-SAMP/Siglec pathway in vitro and in vivo can open the door to increase anti-tumor immunity. Here, this study will discuss the CD33r Siglecs such as Siglec-9 and Siglec-10 along with Siglec-15.

## Siglec-9

Siglec-9 is an inhibitory immune checkpoint receptor of CD33r Siglec family prominently expressed on tumor-infiltrating lymphocytes (TILs) and macrophages [55]. The sufficient binding of sialylated ligands with Siglec-9 typically triggers the upregulation of Sia-SAMPs network that is essential for causing immune evasion and cancer metastasis [56–58]. Therefore, interruption of the Siglec-9/Sia-SAMPs pathway can suggest a therapeutic intervention to improve the effector T cell response against cancer [59]. Stanczak et al. reported the overexpression of Siglec-9 checkpoint receptor on the surface of TILs in the aspect of colorectal, NSCLC (non-small cell lung cancer), and ovarian cancer patients and also observed the co-expression of other inhibitory receptors like PD-1 [60]. It has been already proved that Siglec-9 inhibits NK cell-mediated tumor cell killing in vitro [61]. Again, the binding affinity of macrophage-derived Siglec-9 with sialylated glycoform can activate TAM (tumor-associated macrophage) to escape immune-regulatory mechanisms and further promote cancer progression [56]. Furthermore, Stanczak and his coworkers reported an increase in tumor cell growth due to Siglec-9 upregulation on CD4^+^ and CD8^+^ T cell-bearing mice when compared to control mice in their study. Besides that, when they used two full IgG Siglec-9 antibodies (191,240 and E10-286) to test the effect of Siglec-9 blockade on T cell activation *in vitro*, they found a dose-dependent inhibition of T cell activation [60]. Their study supports the theory that cancer-associated Sia-SAMPs interact with inhibitory Siglec-9 on TILs to promote immune evasion. In addition, the evidence demonstrated by other previous studies also supports their hypothesis in the aspect of tumor immunotherapy. However, it is still not clear what is the exact intracellular signaling pathway that regulates Siglec-9 to inhibit T cell activation. Therefore, the best immune-modulatory functions of Siglec-9 on different immune cells rather than T cells need to be studied further to suggest a potential therapeutic target for treatment.

## Siglec-10

Siglec-10 (sialic acid-binding Ig-like lectin 10) is TAM-expressed CD33 related inhibitory checkpoint receptor of Siglecs family and is considered a promising target for cancer immunotherapy [62]. It is well established that ‘don’t eat me’ signals are naturally generated by cancer cells to evade macrophage-mediated anti-tumor immunity. For example, tumor cells that expressed CD24 antigen, a glycosylated glycosylphosphatidylinositol-anchored surface protein, are capable of producing a ‘don’t eat me’ anti-phagocytic signal to protect cancer cells from macrophage-mediated phagocytosis via its interaction with the Siglec-10 receptor on TAM [51, 63]. Again, the interaction of CD24 protein with Siglec-10 triggers the inhibition of inflammatory responses to liver damage [64], sepsis [65], infection [66], and chronic graft diseases [67]. Hence, the inhibitory signaling pathway is mediated by two SHP-1 and SHP-2 phosphatases that are connected with the cytoplasmic tail of Siglec-10 by using two ITIMs [51, 68]. However, the overexpression of CD24 has been already observed in previous study reports in ovarian and breast cancer cells but it has not yet been declared which is the exact pathway that causes CD24-mediated immune resistance on TAM. Therefore, Barkal et al. used the single-cell RNA sequencing method to test the effects of CD24-Siglec-10 signaling cascade on macrophage-mediated tumor controlling ability within the tumor microenvironment, and they found the high expression of CD24 and Siglec-10 in several tumors that they analyzed at a cellular level which ultimately indicates the potential of CD24 as a tumor-specific marker [63]. Again, the upregulation of macrophage-derived Siglec-10 at a substantial amount was determined by FACS in the aspect of breast and ovarian cancer cells which elicits a strong affinity of CD24 to interact with Siglec-10 receptor. In addition, they obtained the enhancement of anti-tumor immunity by macrophages when monoclonal antibodies were used to block of CD24/Siglec-10 anti-phagocytic signal which strongly supports the Siglec-10 inhibition activity against phagocytosis. Therefore, the knockout of either Siglec-10 receptor, or CD24 protein, and antibody blockade of CD24/Siglec-10 cascade further showed the robust phagocytic expression of macrophages to restrict tumors growth [63]. These types of evidence strongly support that the blockade of CD24/Siglec-10 immune checkpoint pathway might provide an effective therapeutic target to improve anti-tumor immunity in the aspect of breast and ovarian cancer hosts.

## Siglec-15

Unlike Siglec-9 and Siglec-10, Siglec-15 belongs to the conserved gene family with a characteristic sialic acid-binding immunoglobulin type lectin structure [69]. Within the TME, Siglec-15 also contains the same domain composition as the PD-1/B7-H1 immune checkpoint and thereby suppresses immune responses against tumor progression, whereas the activation of suppressed T cells can restore defective immunity through the blockade of PD-1/B7-H1 signaling cascade with monoclonal antibodies which ultimately indicates the evidence of normalization theory in cancer immunotherapy [70, 71]. However, other cellular or molecular mechanisms including the presence of dysfunctional T cells, TAM, and myeloid-derived suppressor compounds, the insufficient infiltration of immune cells, and the downregulation of immunomodulatory cells such as different cytokines and metabolites are critically involved to create immune evasion in TME along with the upregulation of PD-1/B7-H1 pathway based on the reports of numerous studies [72–75]. Hence, the selective target of myeloid cells-derived Siglec-15-mediated immune suppression can improve the therapeutic efficacy of PD-1/B7-H1 blockade strategy which then represents acquired resistance in the aspect of several human cancers [76, 77]. A recent study by Jun Wang’s group represented the suppression of T cells activity *in vivo* and *in vitro* via the overexpression of macrophage-derived Siglec-15 in different human cancer cells. In addition, they also found the inhibition of tumor growth in multiple tumor models when they used antibodies to block Siglec-15. Furthermore, they confirmed that the M-CSF (macrophage colony-stimulating factor) induced overexpression of Siglec-15 on tumor-associated macrophages can be downregulated by IFN-γ which is a positive regulator of the B7-H1 family [78]. Collectively, the above results strongly support the potential of Siglec-15 as an immune-suppressive molecule in TME. Therefore, the antibody blockade technique of Siglec-15 expression may be used as an attractive therapeutic approach to target cancer patients who are resistant to current anti-PD-1/B7-H1 therapy [79, 80]. However, the exact pathway that induces the overexpression of Siglec-15 in many cancers and/or tumor-infiltrating macrophages compared to normal tissues is still unknown which can be identified to enhance anti-tumor immunity.

## CD47-SIRPα checkpoint-based immunotherapy

**Fig. 1:**
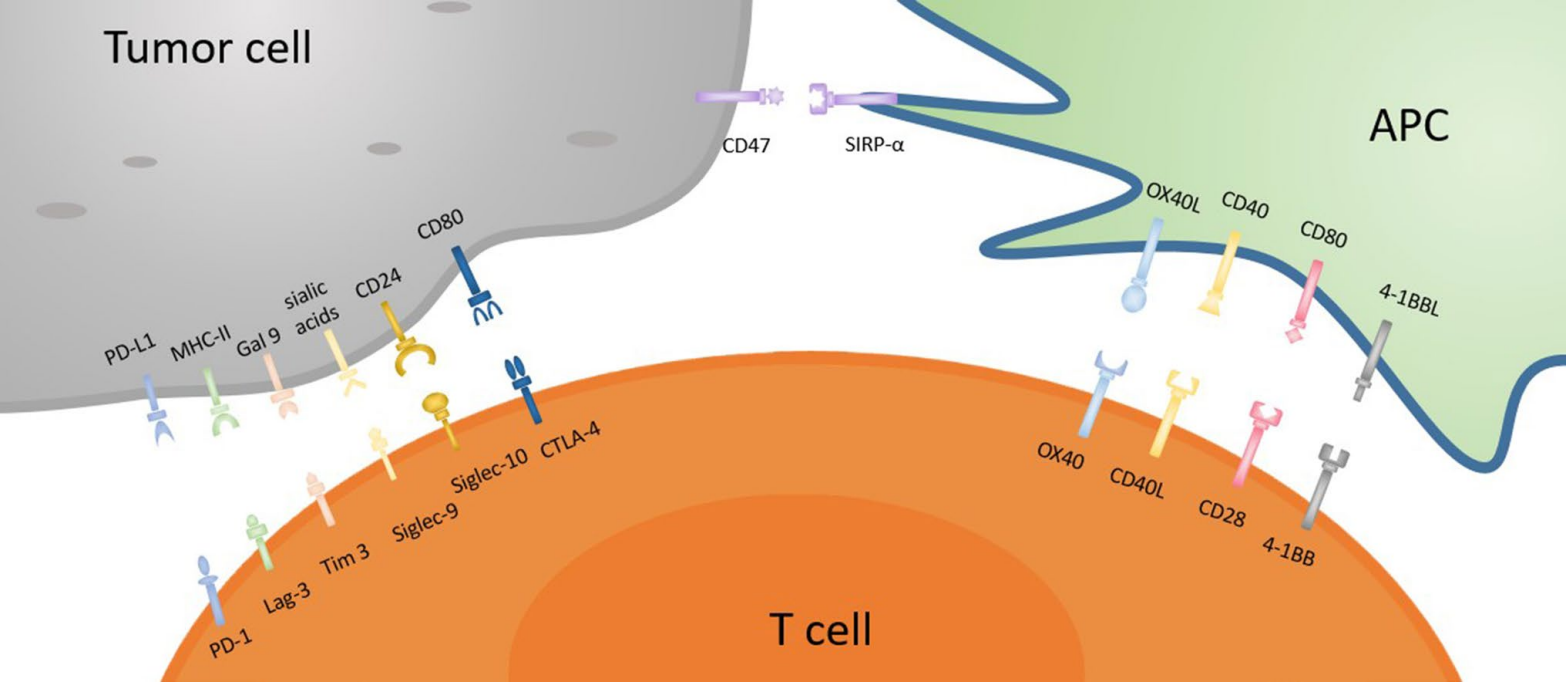
Inhibitory and stimulatory receptors on APC/T cells and tumor cells

CD47-SIRPα checkpoint is a myeloid cell-derived immune checkpoint that also plays an important role in inducing immune evasion of cancer cells like other immune checkpoints [81–83]. Hence, tumor cells express CD47 protein that is then able to bind with SIRPα receptor on myeloid cells such as macrophages and neutrophils [84]. The interaction of the CD47 ligand with the SIRPα receptor finally triggers the cancer cells to develop a ‘don’t eat me’ signal toward the immune response of T cells [85, 86]. Nowadays, this signaling pathway has been studied as a phagocytosis checkpoint [25] and the design of therapeutic strategies to disrupt the CD47-SIRPα axis through clinical investigations is ongoing [87]. However, prior research data strongly suggest that the blocking of CD47-SIRPα interaction with tumor opsonizing antibodies (such as anti-CD20 rituximab, or, anti-EGFR cetuximab, or anti-Her2 trastuzumab) can significantly lead to cancer cell clearance especially in case of liver cancer through suppressing cancer cell-expressed CD47 proteins [86, 88–90]**.** Therefore, by using a haploid genetic screen, Logtenberg and his colleagues identified the presence of Golgi body and endoplasmic reticulum (ER) secreted QPCTL enzyme which belongs to the QPCT (Glutaminyl-peptide cyclotransferase) family and also stated that QPCTL is the critical regulator of the CD47-SIRPα axis. QPCTL is involved in the modification of CD47 protein due to the formation of pyroglutamate (pGU) residue at the N-terminus end of CD47 protein, and thereby it can interact with the SIRPα site [91, 92]. In addition, another hypothesis arises from their study that the deletion or inhibition of QPCTL protein can provide strong evidence of macrophage and neutrophils-mediated tumor cell phagocytosis and cellular toxicity which is antibody dependent. Anyway, they did not show the effect of QPCTL inhibition on T cell responses. Collectively, the obtained results from their research data reveal that the development of novel QPCTL inhibitory molecules may be fused with antibody blockade of CD47 or SIRPα to promote both innate and adaptive immunity. Besides, targeting the CD47-SIRPα signaling cascade along with the existing therapeutic approach such as CTLA-4 and PD-1 may also contribute to the enhancement of immunotherapy efficacy in the future (Fig. 1).

## Intrinsic molecular pathway based immunotherapy

Adoptive T cell therapy (ACT) has brought out unprecedented clinical outcomes in cancer immunotherapy as dysfunction of effector T cell is considered the central point for causing immune evasion by cancer cells. However, the main drawback of this method is the short-term immune response of adoptively transferred engineered T cells in some cancer patients [93, 94]. Therefore, to enhance the therapeutic efficacy of ACT, it is further required the transformation of CD8^+^ T cells into long-lived effector T cells by targeting some regulatory proteins those act as T cell suppressors or activators in TME, including REGNASE-1, PAC1, and AGK [95].

### REGNASE-1

REGNASE-1 is a major negative regulator of anti-tumor response mediated by T cells which are encoded by the Regnase 1 gene (also known as zc3h12a) with having RNA degrading properties) [96, 97]. The exact role of its in TME is still not clear due to poor study of the fate of T cells although it has shown crucial effects on B cell homeostasis [98]. Jun and his group revealed that normal CD8^+^ T cells can be reprogrammed into effector T cells with long-term immunity by using the knowledge of CRISPR/cas9 mutagenesis approach for REGNASE-1 block which controls T cell activation. Again, they identified BATF as a key controlling agent of REGNASE-1 and confirmed the enhancement of therapeutic efficacy of REGNASE-1 deficient CD8^+^ T cells when they targeted BATF along with two other essential factors such as PTPN2 and SOCS1 by using genome-scale CRISPR screening technique [99, 100]. However, their findings ultimately suggest that targeting CD8^+^ T cell-expressed REGNASE-1 with other immune checkpoint inhibitors may also open the door to new therapeutic approaches to provide effective anti-tumor immunity by CRISPR method.

### PAC1

PAC1 (Phosphatase of activated cell 1) is another negative immune checkpoint typically involved in the attenuation and exhaustion of CD8^+^ T cell-mediated anti-tumor immunity [101, 102]. The upregulation of PAC1 has selectively occurred in exhausted TIL which is responsible for the poor prognosis of cancer patients [103]. Recently, the most promising approach to reprogramming exhausted T cell into effector T cell is combining checkpoint-based therapeutic strategies with chemical pathway as oxidative stresses such as ROS plays an important role to control T cell immune response against cancer [48, 104]. Thus, it is needed to detect the specific pathway by which effector T cell activity is hampered. Besides, from the previous study, it has been already shown that excessive ROS production can exacerbate T cell exhaustion via the suppression of IL-17 and type-1 interferon production [105, 106]. However, the direct way by which oxidative stress compounds regulate CD8^+^ T cell-specific gene expression is still elusive. Furthermore, Dan lu and his team found that the activation of EGR1 (redox transcription factor) mediated by oxidative stress generally induces the expression of PAC1 at a high rate in CD8^+^ T cells within the tumor microenvironment. Next, the upregulation of PAC1 triggers the recruitment of Mi-2β NuRD (nucleosome-remodeling-deacetylase) complex that is responsible for causing alteration of effector T cell genes expression, thereby ultimately leading to inactivation of T cell and further tumor progression [103]. Moreover, their research data collectively support that PAC1 serves as a T cell-specific negative immune checkpoint at the epigenetic level and thus targeting the ROS-EGR1-PAC1 signaling pathway may be a potential target of chemical-based immunotherapy which could offer an alternative way to boost the efficacy of T cell immune response in terms of cancer treatment and prevention.

### Acylglycerol kinase (AGK) pathway

AGK (Acylglycerol kinase) is a positive regulator of CD8^+^ T cells glycolysis (glucose metabolism) which is a prerequisite for effector CD8^+^ T cells proliferation and activity, while CD8^+^ T cells play a pivotal role in adaptive anti-tumor immunity to eliminate cancer cells [107]. Indeed, AGK is an enzyme of lipid kinase property that is involved in the conversion of acylglycerol to lysophosphatidic acid (LPA) through phosphorylation and also regulates tumor expansion [108]. Bektas et al. reported that AGK is involved in the regulation of EGF (epidermal growth factor) signaling in prostate cancer cells [109]. Although it has been established from previous data that glycolytic metabolism plays a crucial role in the activation and functional fitness of CD8^+^ T cells, but the exact molecular mechanism that regulates the glycolysis of CD8^+^ T cells is still elusive. However, to unravel the AGK-mediated elevated CD8^+^ T cell glycolysis, Hu and his colleagues performed several *in vivo* and *in vitro* studies and it has been reported from their research data that AGK can also act as a protein kinase in the glycolytic metabolism of CD8^+^ T cells through the regulation of PTEN activity. Hence, PTEN is regarded as an inhibitor of PI3K (phosphatidylinositol-3-OH kinase)-mTOR (mammalian target of rapamycin) signaling which typically controls the metabolic switch of CD8^+^ T cell upon stimulation of TCR and CD28 molecules and that is why it is also known as an AGK-regulated checkpoint [110, 111]. At a molecular level, they found that the stimulation of TCR and CD28 induces the recruitment of PTEN in the plasma membrane, and then, AGK-PTEN interaction triggers the phosphorylation of PTEN (at Ser380, Thrc382, and Thr383 positions) which restricts the phosphatase activity of PTEN, thereby promoting the activation of PI3K-mTOR signaling cascade and glycolysis of CD8^+^ T cells. On the other hand, the absence of AGK in CD8^+^ T cells showed the impairment of glycolysis and effective anti-tumor functions *in vivo* and *in vitro* respectively. Collectively, these findings demonstrate that AGK-mediated activation of the PI3K-mTOR pathway mainly contributes to the metabolic programming and functional fitness of CD8^+^ T cells [112, 113]. Therefore, targeting the activity of AGK in CD8^+^ T cells may provide a better promising approach to promote the efficacy of T cell-mediated adaptive immune response.

## NK cells-derived immune checkpoint-based immunotherapy

Even though T cell-based checkpoint immunotherapy has revolutionized cancer treatment, the poor immunogenic response of T cell-based immune checkpoint strategies against CTLA-4 and PD-L1 triggers the discovery of novel ‘checkpoints’ on other immune cells, particularly natural killer (NK) cells, which will be targeted in the future [114–116]. NK cells are the essential components of innate immunity that can release cytotoxic granules, such as perforin and granzymes to destroy cancer cells [115, 117], or can cause cancer cell apoptosis through the production of TNF-α, FasL, and TRAIL genes [118, 119]. The effector function of NK cells-mediated cytotoxicity is not only well recognized against blood tumors and metastatic tumors state but also significant patient outcomes have been confirmed in several solid tumors such as colon, lung, stomach cancer, and renal carcinoma [120], whereas the dysfunction of NK cells via knockout of IFN-γ or perforin genes has already shown a higher efficacy of carcinogenesis in mouse models of metastasis [121]. On the other hand, for enhancing the CD8^+^ T cells-mediated anti-tumor response, NK cells play an important role to facilitate the accumulation of cDC1 (conventional type of dendritic cells) into cancer cells via the production of chemo-attractants such as CCL5 and CXCL1 [122]. In addition, NK cells are generally able to eliminate the tumors that are not detected by the T cell immune-surveillance system due to the reduced expression of MHC-1 molecules. These promising features of NK cells highlight that they can be used as an emerging target of cancer immunotherapy along with boosting the efficacy of T cell-mediated anti-tumor response [123]. However, the effective anti-tumor function of NK cells mainly depends on the integration of some immune-activating and immune-suppressive signals/pathways. Among them, different types of extrinsic and intrinsic immune inhibitory receptors such as KIRS (killer inhibitory receptors), CD96, TIGIT, PD-1, CTLA-4, TIM-3, VISTA, and LAG-3, also known as checkpoint receptors, are responsible to prevent the tumor-killing potentiality of NK cells through their upregulation on NK cell surface which is typically mediated by IL-10 and TGF-β [120]. In this review, we will shed light on the brief discussion of the targeting strategy of NK-based checkpoint receptors especially CD96 and NKG2A receptors with monoclonal antibodies to exploit the potential anti-tumor activity of NK cells as a helper of T cell-based checkpoint immunotherapy.

### CD96

CD96, a transmembrane checkpoint receptor of the immunoglobulin family, is normally expressed on the surface of NK cells and T cells and was previously shown to promote NK cell-mediated cancer cell cytolysis [124]. The deficiency of NK cells-derived CD96 checkpoint was showed increased tumor resistance in chemically produced tumor models of mice which were also dependent on NK and IFN-γ [125]. In contrast to NK cells from peripheral tumors, tumor-infiltrating NK cells had a higher proportion and intensity of CD96 on them, as well as a higher quantity of CD96^+^ NK cells in HCC patients. Later, the increased IL-10 and TGF-β production and the decreased IFN-γ and TNF-α production confirmed the dysfunction of CD96^+^ NK cells more than CD96^−^ NK cells. Interestingly, the higher expression of CD96 or its ligand CD155 resulted in poor clinical benefits in HCC cancer patients (H. Sun et al., 2019) [113]. However, the blockade of CD96 with monoclonal antibody further supports being a checkpoint receptor expressed on NK cells and the efficacy relies on the presence of CD226 or IFN-γ genes. Moreover, the combination of the CD96 blockade strategy with anti-CTLA-4 and anti-PD-1 immunotherapy demonstrated more significant effects to facilitate the infiltration of NK cells into tumor mass [120, 126], which ultimately indicates that targeting of the CD96 checkpoint on NK cells might provide a potential immunotherapy strategy to enhance NK cells-mediated anti-tumor efficacy [127].

### NKG2A

**Fig. 2:**
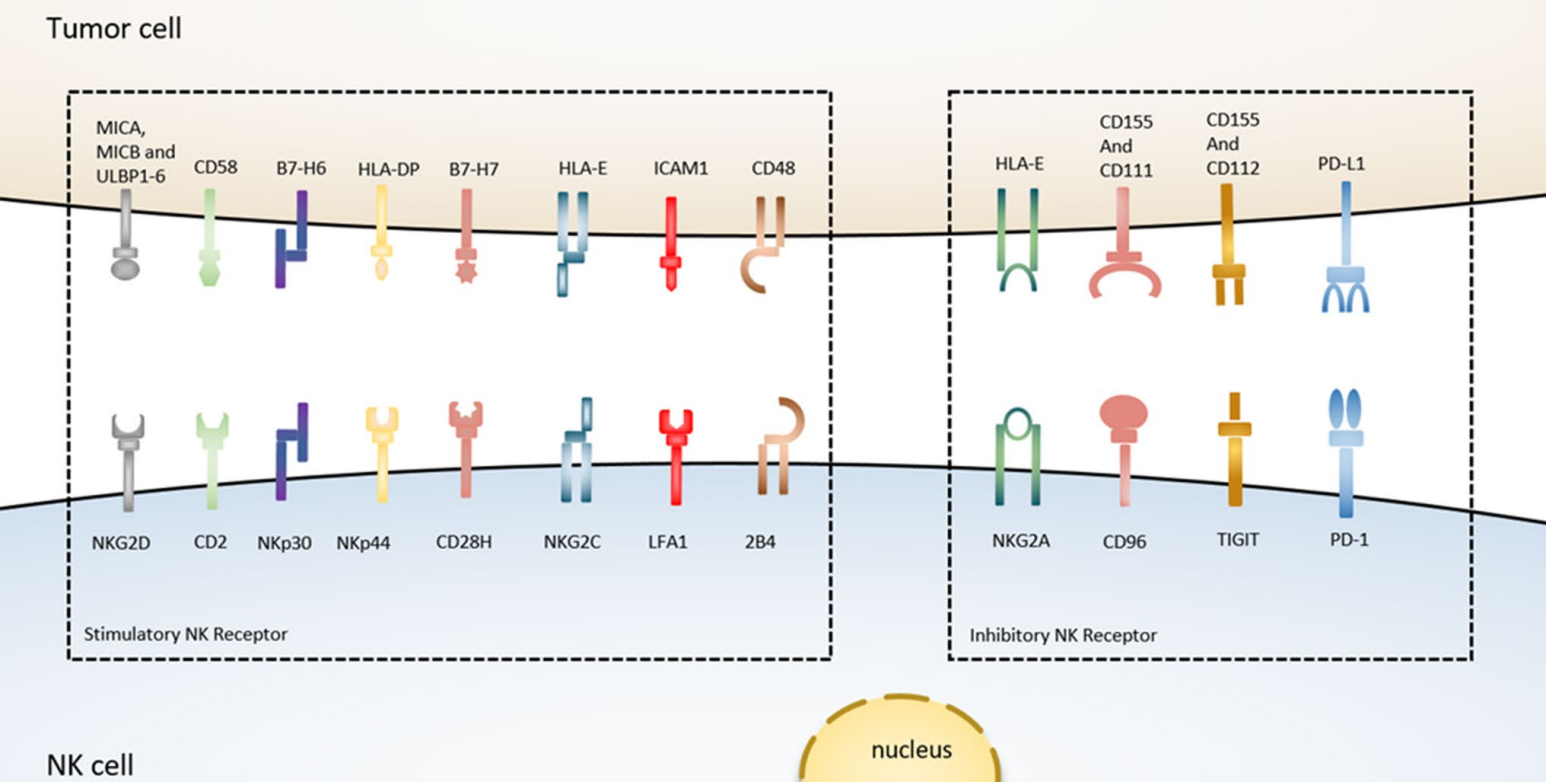
The interaction between NK cells and tumor cells

NKG2A is another checkpoint receptor that is present on both NK cells and T cells and also binds with CD94 to form a heterodimer [128]. In human blood and SCCHN (squamous cell carcinoma of the head and neck) tumors, the amount of NKG2A^+^ NK cells is found as more than half of the quantity. Again, the stimulation of IL-15 further triggers the upregulation of NKG2A on the surface of NK cells [129], and importantly the expression of NKG2A subset along with PD-1 is also found among the NKG2A^+^ NK cells. When NKG2A/CD94 binds to its ligand such as MHC-I and HLA-E (the ligand of NKG2A in humans), such kind of interaction reduces the effector anti-tumor activity of both NK cells and T cells via recruiting SHP-1 tyrosine phosphatase in the intercellular domain of NKG2A. However, blocking the expression of NKG2A reversed the HLA-E-mediated reduction of cytotoxicity and IFN-γ production by NK cells *in vitro*, and increased the efficacy of NK cells against HLA-E tumors *in vivo* after infusion [120, 130]. Besides, the expression of NKG2A and HLA-E in hepatocellular carcinoma (HCC) tissues corresponded with poor prognosis in HCC patients, confirming these functional investigations [48]. Furthermore, the combined therapeutic blockade of NKG2A by monalizumab (a humanized anti-NKG2A antibody) with PD-1/PD-L1 demonstrated the improvement of NK cells-mediated cytotoxic activity and also increased immune response of CD8^+^ T cells against different tumors in compare to PD-1/PD-L1 blockade alone [129]. Collectively, this evidence indicates that the blockade of NKG2A can also stimulate the CD8^+^ T cells-dependent anti-tumor immunity [131], and therefore, it can be considered a promising immunotherapy approach to boost the effector functions of both NK cells and CD8^+^ T cells against the tumor (Fig. 2).

## Targeting TAM to improve the therapeutic effectiveness

In tumor immunotherapy-based research, several clinical and preclinical tests are currently being conducted by combining the pre-existing therapies with novel therapies to get better outcomes for the improvement of immune system efficacy [132, 133]. However, due to the immunogenic properties and microenvironment composition of the tumor, all techniques do not suit all tumors and the response of patients to different approaches also varies based on cancer subtype. For example, the administration of immune checkpoint-based antibodies against PD-1 or CTLA-4 receptors/ligands have shown dramatic effects in melanoma and lung cancer patients [134]. However, this therapeutic strategy does not fully respond to other types of solid tumors, such as pancreatic and breast cancer due to insufficient mutation that hinders the recognition of neo-antigens or tumor cell surface expressing antigenic peptide molecules by CD8^+^ T cells [135].

Solid tumors are formed because of non-hematopoietic stem cell mutations, and finally, they differentiate into malignant forms to cause cancer. In response to solid tumors, myeloid cells-derived immune cells such as regulatory T cells and tumor-associated macrophages (TAM) suppress the tumor-killing nature of cytotoxic lymphocytes such as CD8^+^ T cells and natural killer cells [136, 137]. However, the preferential accumulations of tumor-associated macrophages on the surface of solid tumors limit the cytotoxic potentiality of CTL *in vitro* through either the direct expression of immune-suppressive molecules or indirect recruitment of Treg cells [138–140]. DeNardo found that the increasing anti-tumor immunity mediated by CD8^+^ T cells is mainly due to the depletion of TAM in a mouse model of breast cancer under chemotherapy treatment [141]. Therefore, TAM can be recommended as one of the vital therapeutic targets to increase the potentiality of immunotherapy. Besides, an effective therapeutic technique can be outlined by combining the immune-suppressive molecules along with CTL delivery within the tumor microenvironment. Recently, three therapeutic approaches such as depletion, reprogramming, and molecular targeting have been suggested to recover TAM-mediated immune suppression. It has already been proved that CCR2 triggers the differentiation of circulating monocytes into TAMs in the tumor microenvironment and another receptor that is required for the recruitment, differentiation, and survival of macrophages is called colony-stimulating factor-1 receptor (CSF1R) [142]. In the pancreatic mouse cancer model, the combination of CCR2 antagonist with anti-PD1 antibody therapy reduces the tumor growth, while single anti-PD1 antibody-mediated therapy is not successful [143]. Again, treatment with CSF1R antagonists (e.g., PLX3397, PLX73086, and BLZ945) also significantly decreases the number of TAMs in pancreatic, breast, cervical, mesothelioma, and ovarian mouse cancer models. Furthermore, immune checkpoint inhibitors-mediated blockade of CCR2 or CSF1R receptors can increase the efficacy of immune checkpoint therapy via the prevention of TAM infiltration into tumor [144–146]. Different *in vitro* studies on macrophages have already revealed that pro-inflammatory cytokines are released during the culture of IFN-γ and LPS. This type of cytokine then can help to become immune-activating TAMs from tumor suppressive form in response to certain environmental conditions [147, 148], and thereby can enhance the efficacy of immunotherapy strategy.

Research carried out by Cassetta and Kitamura also supports that the efficacy of cancer vaccination is strongly related to the accumulation and activation of myeloid cells, specifically TAMs. Furthermore, it is also reported in their study that the efficacy of cancer vaccination can be improved by co-injection of immune adjuvants to enhance the host immune responses toward the toll-like receptor (TLR) ligands and DC-targeted antibodies. Additionally, TAM depletion can improve the effectiveness of the therapeutic vaccine with appropriate adjuvants [135]. In TAM reprogramming that mediates immune activation by activated CD8^+^ T cells, depletion of macrophages reduces the effectiveness of therapeutic cancer vaccination, and in this case, TAM targeting techniques need to be carefully combined [149]. For targeting the non-immunogenic cancer cells, the adoptive transfer of genetically engineered CD8^+^ T cells has been emerged as an attractive approach to exert effective tumoricidal ability via the expression of chimeric antigenic receptors (i.e., CAR-T cells). However, the tumor-killing activity of the chimeric antigen receptor (CAR) is restricted by TAM [150–152]. Genetic modulation of CAR-T cells to produce interleukin-12 (IL-12) has also been reported for increasing the effectiveness of immunotherapy via reprogramming of TAM which can express tumor necrosis factor-α (TNF-α) [153]. In the ID8 ovarian cancer model, TAMs found in ascites tumors express a large number of folate receptor β (FRβ) and thereby adoptive transfer of FRβ-specific CAR-T cells into the tumor-bearing mice results in in vitro enhanced cytotoxicity of NK cells [154]. These findings indicate that TAM depletion may increase the efficacy of NK cell infusion therapy (e.g., CCR2 or CSF1R antagonist therapy) by blocking TAM recruitment or survival signals (e.g., the cytotoxicity of CAR-T cell against tumors) [123]. Although the exact molecular mechanism by which TAM inhibits T cell-mediated tumor immunity is still not clear and collectively these studies indicate that either targeting TAM-derived suppressive molecules, or TAM depletion or changing the differentiation pattern can effectively eliminate TAM-mediated restriction of immunity.

## Concluding remarks and future directions

For the treatment of cancer, numerous promising immunotherapy approaches have been emerged by targeting TME in the last few decades based on the knowledge of tumor-induced immune evasion. To escape *T*_eff_ cell-based adaptive immune response, tumor cells continuously change their genetic materials and can express several suppressive molecules which ultimately to dampen immune response. Therefore, to achieve better clinical outcomes in different cancer patients, adaptive immune resistance-based immune evasion must be overcome in future studies. However, the FDA approval of anti-CTLA4 or anti-PD1 checkpoint strategy typically suggests that there is an urgent need to combine them with other therapeutic approaches such as cancer vaccines, different cytokines, TAM and TLR agonists to boost the efficacy of endogenous adaptive immune response against particular tumor sites without any complications or side effects. Basically, in the future newly designed immunotherapy techniques should not only focus on the enhancement of anti-tumor immunity but also need to identify the exact defects of each tumor so that malignant tumors can be transformed into a normalized state with no IREAs (immune-related adverse events) through using selective modification. Furthermore, the principles of different ICIs in the current studies will drive the development of more effective combinatorial tumor immunotherapy strategy by allowing the combinations of T cell-based checkpoint pathways with NK-derived immune checkpoint pathways against poorly immunogenic tumors. Besides, the understanding of cellular and molecular mechanisms of immune checkpoint-based inhibitory pathways may also provide better insight to target different cytotoxic factors such as IL-10, IL-15, VEGF, and TGF-β as well as Siglec-based checkpoints along with T cell and NK-based checkpoint inhibitors for an effective anti-tumor response. Again, to overcome adaptive resistance, ICIs or ACT could be combined with monoclonal antibodies based on specific tumor antigens that will block the functions of immune-suppressive cells including TAMs, Treg, MDSCs, etc. Hence, these types of combination-based immunotherapies may maximize the clinical efficacy in a subset of tumor patients with a long-lasting anti-tumor response rate. Moreover, targeting ICIs with phagocytosis checkpoints such as the CD47-SIRPα axis as well as epigenetic checkpoints like REGNASE-1, PAC-1, or PI3K-AGK pathways which are upregulated in some cancer cells may build up a strong connection between innate and adaptive immune cells to provide a more successful way for the treatments of cancer patients rather than conventional therapeutic approaches. Finally, for improving the combination-based therapeutic efficacy, promising biomarkers may be designed to predict specific tumor response rates for specific treatment strategies based on the disease history of cancer patients. Although our current review discusses the combination-based novel immune checkpoint strategies, a lot of further investigations about the regional properties of different immune cells will need to be unraveled in the future.
